# Material Extrusion of Helical Shape Memory Polymer Artificial Muscles for Human Space Exploration Apparatus

**DOI:** 10.3390/polym14235325

**Published:** 2022-12-06

**Authors:** Kellen Mitchell, Lily Raymond, Joshua Wood, Ji Su, Jun Zhang, Yifei Jin

**Affiliations:** 1 Department of Mechanical Engineering, University of Nevada Reno, Reno, NV 89557, USA; 2 Advanced Materials and Processing Branch, NASA Langley Research Center, Hampton, VA 23681, USA

**Keywords:** artificial muscle, material extrusion, shape memory polymer, operating conditions, phase diagram

## Abstract

Astronauts suffer skeletal muscle atrophy in microgravity and/or zero-gravity environments. Artificial muscle-actuated exoskeletons can aid astronauts in physically strenuous situations to mitigate risk during spaceflight missions. Current artificial muscle fabrication methods are technically challenging to be performed during spaceflight. The objective of this research is to unveil the effects of critical operating conditions on artificial muscle formation and geometry in a newly developed helical fiber extrusion method. It is found that the fiber outer diameter decreases and pitch increases when the printhead temperature increases, inlet pressure increases, or cooling fan speed decreases. Similarly, fiber thickness increases when the cooling fan speed decreases or printhead temperature increases. Extrusion conditions also affect surface morphology and mechanical properties. Particularly, extrusion conditions leading to an increased polymer temperature during extrusion can result in lower surface roughness and increased tensile strength and elastic modulus. The shape memory properties of an extruded fiber are demonstrated in this study to validate the ability of the fiber from shape memory polymer to act as an artificial muscle. The effects of the operating conditions are summarized into a phase diagram for selecting suitable parameters for fabricating helical artificial muscles with controllable geometries and excellent performance in the future.

## 1. Introduction

The zero-gravity and/or microgravity environments astronauts must endure are made incredibly physically demanding through skeletal muscle atrophy, which is caused by leaving Earth’s gravitational field. Muscle strength and articulation are imperative for astronauts’ effectiveness and safety when aboard the International Space Station (ISS), traveling through deep space, or adjusting to the gravity of terrestrial bodies beyond Earth [1]. To help reduce skeletal muscle atrophy on the ISS, astronauts are allotted 2.5 h of exercise per day using specialized equipment [2]. 

Exoskeletons can be used as muscular assistive devices for astronauts during human space exploration in strenuous situations to mitigate risk. Using actuators, linkages, and pivots, exoskeletons aid the wearer’s muscles, bones, and joints [3,4,5]. In recent years, research in exoskeletal design has replaced electric motors with artificial muscles to lower the device’s weight, resulting in less physical demand on the user [4,5]. Pneumatic artificial muscles use compressed air to fill ribbed pneumatic chambers or balloon structures within a textile sleeve to act in concert with the wearer’s body [6,7]. Despite their advantages, exoskeletons using pneumatic artificial muscles require heavy pumps to drive the system and compressed air that could be better allocated for sustaining astronauts, making them costly for transport and resource inefficient for human space exploration. Shape memory alloy (SMA) [8,9] and shape memory polymer (SMP) [10,11,12,13,14,15,16,17,18,19,20,21,22] based artificial muscles do not require heavy motors or pumps to actuate. Instead, shape memory materials use external stimuli, such as temperature, magnetic field, or pH level changes, to alter the shape and/or mechanical properties of a structure. Generally, SMA materials are more complex to design, weigh 5–7 times more, and are costlier to manufacture than SMPs [23,24,25]. Therefore, artificial muscles from SMPs are more popular and promising for aerospace applications. 

Spinning, twisting, and three-dimensional (3D) printing are the most widely used manufacturing techniques for producing SMP artificial muscles. Three subcategories of spinning are used to fabricate artificial muscles: melt spinning, wet spinning, and electrospinning. In melt spinning, viscous polymer melt is drawn from a spinneret, cooled, and spooled onto a rotating drum, resulting in a long, single polymer fiber [10,11,12]. To produce polymer fibers through wet spinning, polymer powders are dissolved in a solvent and then extruded through a spinneret into a solvent/non-solvent, where the polymer coagulates to form a fiber [13,14]. Lastly, SMP artificial muscles can be produced through electrospinning by ejecting a polymer solution from a conical nozzle using an electric charge, resulting in strands of polymer fibers [15,16]. Spinning can produce SMP artificial muscles rapidly; however, all three methods can only produce fibers with small, thread-like geometries, which limits the contraction force and requires large arrays of SMP fibers for a single muscle actuator. Furthermore, the spinning devices and processes are too complex to be performed by astronauts aboard a space shuttle or the ISS [26]. Twisted artificial muscles combine the effectiveness of spun SMP materials with the contraction strain advantage of a helical geometry. To form artificial muscles by twisting, nylon fibers are twisted at different tensions to form twisted fibers. Then, the twisted fiber is either coiled around other twisted fibers or a mandrel to generate supercoiled polymers (SCPs) [18]. Lastly, the fibers are temperature annealed to hold a desired helical shape. After fabrication, when heat is applied to twisted SMP artificial muscles through joule heating or convection, the fibers contract because of the material’s anisotropic thermal expansion and helical shape [17,18]; generating a pulling force. Like spinning, producing SMP artificial muscles through twisting requires highly trained operators for fabrication and multiple machines for twisting and annealing, which may occupy the limited room within a spacecraft. The development of 3D printing techniques, specifically material extrusion methods, provides an alternative strategy to fabricating helical SMP artificial muscles. There are three subcategories of material extrusion 3D printing: fused deposition modeling (FDM), direct ink writing (DIW), and support bath-assisted 3D printing. In FDM and DIW, a printhead selectively deposits a thermoplastic filament or self-supporting ink layer onto a planar or cylindrical substrate. By applying different crosslinking mechanisms, such as temperature change or ultraviolet radiation (UV), the deposited layer is solidified with the enhanced mechanical stiffness necessary to support the subsequent layers. Thus, complex 3D structures, such as helical SMP artificial muscles, can be built layer-by-layer by moving the printhead vertically between layers [19,20,21,22]. To print artificial muscles through FDM, support materials must be created in the overhanging sections of the helix profile, resulting in large quantities of material waste [27]. Because self-supporting inks are used in DIW, support structures are not necessary. However, the material selection of SMPs is severely limited to UV crosslinkable polymers or polymer solutions with rapid curing speeds. SMP artificial muscles have been printed via support bath-assisted 3D printing [28,29], in which polymer filament is directly printed into a helical shape within a liquid support bath. Although the support bath can hold the printed artificial muscle in situ, making it unnecessary to print support structures, it also increases the number of materials needed to produce an artificial muscle as well as the weight of the printing device, resulting in a cost-ineffective fabrication strategy for human space exploration. Additionally, all previously mentioned 3D printing methods require a three-axis gantry and substrate to fabricate artificial muscles, further increasing the weight and volume of the system. As a result, it is of great significance to investigate a new strategy that forgoes the need for a gantry system and substrate with the ability to fabricate helical SMP artificial muscles for human space exploration. 

Recently, a material extrusion system [30] has been designed and fabricated to manufacture helical artificial muscles. It uses an internal, helical fluted mandrel to extrude SMP melts and an accurately controlled temperature gradient to cool and stereotype molten SMP as it exits the printhead. Since it is easy to operate and does not require a gantry, substrate, or support bath during fabrication, this method is promising for spaceflight. However, only one current study exists validating the effectiveness of the system, and it lacks exploration on the effects of operating conditions, such as the printhead temperature, cooling fan speed, and inlet pressure, on the helical geometry as well as the mechanical and shape memory properties. In this study, key geometries of the artificial muscles printed at various operating conditions have been systematically evaluated. Additionally, a phase diagram has been constructed to guide the selection of suitable operating conditions. Finally, the mechanical properties, surface morphology, and contraction stress of the extruded artificial muscles have been assessed to determine their potential and functionality as actuators in exoskeletal devices for deep space missions.

## 2. Materials and Methods

### 2.1. Materials

Luminy LX175 polylactic acid (PLA) pellets (TotalEnergies Corbion, Amsterdam, The Netherlands) were selected as the SMP build material for all helical artificial muscles in this study. This particular PLA has a density of 1240 kg/m^3^, glass transition temperature of 55 °C, melting temperature of 155 °C, and thermal conductivity of 0.132 W/m·K [31].

### 2.2. Single-Factor Experiment Design

The representative helical artificial muscles in this study were printed using the same extrusion system and process described by our previous research [30]. Printhead temperature, cooling fan speed, and inlet pressure were determined to be key extrusion conditions because of their direct effect on the build material cooling and flow rates. Therefore, 13 experiments were designed in which one of the key extrusion conditions is varied while the other two conditions are held constant to evaluate the effects of the conditions on helical muscle formation. Printhead temperatures of 120 °C, 125 °C, 130 °C, and 135 °C and inlet pressures of 10 psi, 12 psi, 14 psi, 16 psi, and 50 psi were tested. Additionally, cooling fan speeds of 25%, 50%, 75%, and 100% of the cooling fan max speed of 11.7 m/s were also tested. When printhead temperature, cooling fan speed, or inlet pressure are not varied, they are constant at 125 °C, 75% of the max fan speed, and 12 psi, respectively. The 13 experiments and resulting muscle groups are shown in Table 1, where three muscles were printed for each combination of extrusion conditions. Consequently, the experiment design resulted in repeat extrusion conditions for specific muscle groups; therefore, muscle groups 2, 7, and 10 have identical extrusion conditions and thus share the same three printed muscles and experimental results. For simplicity, when discussing muscle groups 2, 7, and 10, muscle group 2 will be referenced. During printing for all artificial muscles, the material reservoir temperature was held at a constant 215 °C to ensure all PLA pellets were molten and homogeneous upon entering the mandrel within the printhead. Lastly, an internal printhead mandrel with a flute diameter, depth, and pitch of 4 mm, 1 mm, and 8 mm, respectively, was used to fabricate all muscles.

### 2.3. Helical Geometry Measurement

The average outer diameter, fiber thickness, and pitch of each muscle group were measured using a high-precision measurement system (Vertex 261 Micro-Vu, Windsor, CA, USA) and metrology software (InSpec 2.104.3, Micro-Vu, Windsor, CA, USA). The averaged geometries across all muscle groups were then evaluated against each other to determine how extrusion conditions affect the helical geometry of the muscles. The average geometries are also compared to the corresponding flute diameter, depth, and pitch of the mandrel to determine which extrusion conditions result in geometries that closely resemble the mandrel.

### 2.4. Rheological Property Testing

Shear rate sweeps were performed at 160 °C to investigate the zero-shear rate viscosity just above the melting temperature of the PLA build material. The viscosity was measured at shear rates between 0.01 s^−1^ and 10 s^−1^ and fit to the Carreau model to determine the zero-shear rate viscosity using an Anton Paar rheometer (MCR 92, Anton Paar, Graz, Austria). The rheology tests were conducted using a 25 mm diameter parallel plate tool at a tool gap of 1 mm.

### 2.5. Mechanical Property Testing

The mechanical properties of the artificial muscles were characterized through tensile tests using a mechanical property tester (eXpert 7600; ADMET, Norwood, MA, USA). Muscle groups 2 and 6 were used as representative artificial muscles to evaluate the effects of extrusion conditions on mechanical properties. The artificial muscles were tensioned along the helical axis at a strain rate of 25 mm/min. The average cross-sectional area was calculated for each muscle group tested using the measured geometries of the artificial muscles. Load and displacement data from testing, along with the calculated average cross-sectional area, were used to construct respective stress–strain curves and calculate elastic moduli for the respective muscle groups.

### 2.6. Surface Morphology

The previously described high-precision measurement system and inspection software were used to inspect the surface morphology and measure the surface roughness of the helical artificial muscles. Muscle groups 2 and 6 were selected as the representative artificial muscles to compare how extrusion conditions affect surface morphology and roughness. The arithmetic mean surface roughness was measured on the linear portions of the artificial muscles using the InSpec metrology software. In total, 100 equally spaced points were fitted across 2 mm of the artificial muscle edge at linear sections using the measurement software. The locations of the fitted points were used to calculate a mean line and the arithmetic mean of all 100 points from the mean line, resulting in a surface roughness value for a section of the muscle.

### 2.7. Contraction Testing

Muscle group 2 was also selected as the representative artificial muscles for contraction testing. To perform contraction tests, each artificial muscle was programmed at the glass transition temperature (55 °C) of the SMP material, cooled to harbor the necessary internal stress that causes contractions, and reheated above the glass transition temperature to generate contractions. To begin programming, an artificial muscle is heated to 55 °C for five minutes using a 150-watt heat lamp (Feit Electric, Pico Rivera, CA, USA). Then the muscle is elongated to a strain of 10% using the previously described material property tester while still under heat. The artificial muscle was removed from heat and allowed to cool back down to room temperature at the elongated state. Finally, the muscle was reheated to 60 °C to induce contractions. During reheating, the mechanical property tester was used as a load cell to measure the contraction stresses of the artificial muscle over time. From the contraction stress data, an average max contraction stress and stress rate were calculated for the representative artificial muscles.

### 2.8. Artificial Muscle Length and Statistical Analysis

All artificial muscles were trimmed to a target length of 75 mm for all measurements and tests. During trimming, the tail end of the muscle was removed to reach the target length. All quantitative values of artificial muscle measurements in the text and figures were reported as means ± standard deviation (SD) with *n* = 3 samples per group.

## 3. Results and Discussion

### 3.1. Helical Artificial Muscle Extrusion Mechanism

**Figure 1:**
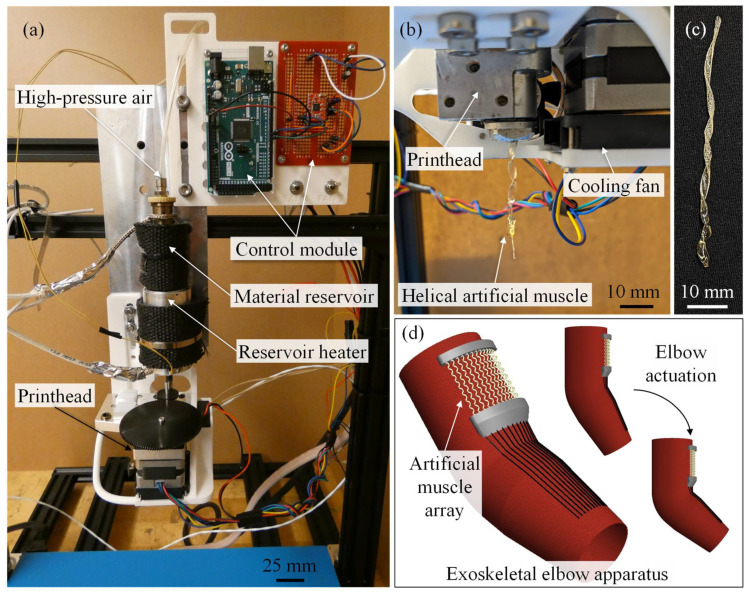
(**a**) Artificial muscle extrusion system. (**b**) Extrusion system printhead during artificial muscle extrusion. (**c**) Polylactic acid helical artificial muscle. (**d**) Potential helical artificial muscle application driven exoskeletal elbow apparatus.

The extrusion system, shown in Figure 1a,b, uses an internal mandrel design, material reservoir heater, printhead heater, cooling fan, and high-pressure air to extrude helical SMP artificial muscles without the need for a substrate or support materials. By controlling the extrusion conditions (printhead temperature, cooling fan speed, and inlet pressure), molten SMP build material begins to solidify within the printhead. The semi-solid polymer is supported by the helical flute of the mandrel during extrusion allowing for helical shape retention, as shown in Figure 1b. The extrusion process results in a continuous helical SMP structure that can be used as an artificial muscle. To produce helical artificial muscles from the extrusion system, a SMP build material with high extrudability and shape memory properties must be used. Herein, Luminy LX175 PLA was selected as the build material because it possesses good extrudability, and PLA is a well-documented SMP that can be programmed to have linear contractions using heat as the shape memory stimulus [30,32,33]. A typical PLA helical artificial muscle is shown in Figure 1c. Because the muscle is supported by the mandrel, a substrate, support bath, and three-axis gantry system are not required for fabrication, making the device compact and light weight. The extrusion printhead system’s reduced size and weight increases its potential usability for astronauts to produce artificial muscles for exoskeleton devices such as the one shown in Figure 1d.

Not only does the helical mandrel support the muscle as it extrudes, but it also aids in the shape memory effects of some SMP materials. As the polymer melt flows through the mandrel, the polymer chains within the melt align to the longitudinal direction of the helical flute of the mandrel. SMPs with anisotropic coefficients of thermal expansion benefit greatly from this polymer alignment and helical geometry, allowing for larger contraction strains and forces, as well as fewer muscles within an artificial muscle array to produce the desired muscle contractions [17,18,34]. 

### 3.2. Printing Condition Effects on Artificial Muscle Geometry

Consistent and predictable muscle geometry is paramount for artificial muscle production. Varying the key extrusion conditions will either generate condition combinations that successfully extrude muscles of various geometries or conditions that cannot produce proper helical structures. Furthermore, specific combinations of key extrusion conditions may result in different helical geometries using the same internal mandrel. The ability to produce muscles with different geometries from the same mandrel increases the usefulness of the extrusion system by allowing the operator to produce a wide variety of muscle geometries without altering the printhead’s internal mandrel. Therefore, it is critical to discover what ranges of each key extrusion condition produce suitable helical artificial muscles, what combinations produce geometries close to the geometry of the mandrel, and how the conditions affect the overall geometry.

**Figure 2:**
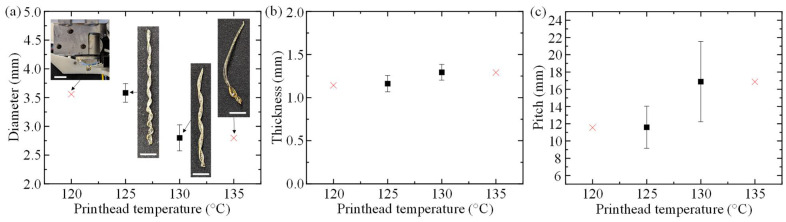
Measured geometry of artificial muscles extruded at a constant cooling fan speed of 75% and inlet pressure of 12 psi. (**a**) Effects of printhead temperature on muscle diameter and representative extrusion results. (**b**) Effects of printhead temperature on fiber thickness. (**c**) Effects of printhead temperature on pitch (scale bar: 10 mm).

**Figure 3:**
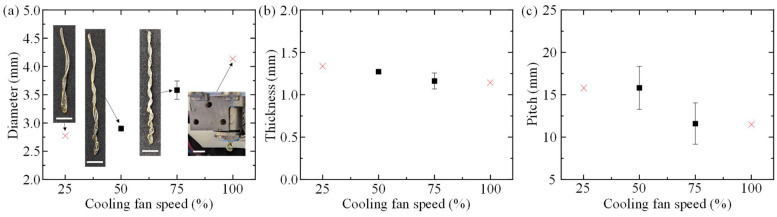
Measured geometry of artificial muscles extruded at a constant printhead temperature of 125 °C and inlet pressure of 12 psi. (**a**) Effects of cooling fan speed on muscle diameter and representative extrusion results. (**b**) Effects of cooling fan speed on fiber thickness. (**c**) Effects of cooling fan speed on pitch (scale bar: 10 mm).

**Figure 4:**
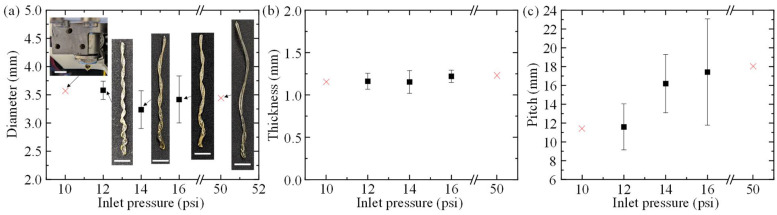
Measured geometry of artificial muscles extruded at a constant printhead temperature of 125 °C and cooling fan speed of 75%. (**a**) Effects of inlet pressure on muscle diameter and representative extrusion results. (**b**) Effects of inlet pressure on fiber thickness. (**c**) Effects of inlet pressure speed on pitch (scale bar: 10 mm).

To determine the effects of extrusion conditions on muscle geometry, the single-factor experiments described in Table 1 were performed, and the resulting artificial muscle diameters, fiber thicknesses, and pitches were measured. The average geometries for the corresponding muscle groups are summarized in Figure 2, Figure 3 and Figure 4, and representative images of the fabricated muscles printed at their unique conditions are shown collectively in Figure 2a, Figure 3a and Figure 4a.

The geometries for muscle groups 1, 2, 3, and 4 are illustrated in Figure 2. When the cooling fan speed and inlet pressure are held constant at 75% and 12 psi, respectively, and printhead temperature increases from 125 °C to 130 °C, the diameter decreases from 3.58 ± 0.16 mm to 2.80 ± 0.23 mm, thickness increases slightly from 1.16 ± 0.09 mm to 1.29 ± 0.09 mm, and pitch increases from 11.59 ± 2.44 mm to 16.89 ± 4.65 mm. The inability to extrude or form the desired helical shape at 120 °C and 135 °C, respectively, is also shown in Figure 2a. When the printhead temperature is set to 120 °C, the molten PLA within the system solidifies rapidly and terminates flow within the flute of the mandrel, effectively clogging the printhead. On the other hand, if the printhead temperature is set to 135 °C or above, the PLA cannot cool significantly inside the printhead to retain the helical shape upon exit, resulting in a non-helical extrusion.

For muscle groups 5, 6, 2, and 8, helical geometry measurements are shown in Figure 3. When the printhead temperature is held at 125 °C, inlet pressure is a constant 12 psi, and the cooling fan speed is increased from 50% to 75%, the artificial muscle outer diameter increases from 2.90 ± 0.01 mm to 3.58 ± 0.16 mm, thickness decreases slightly from 1.27 ± 0.02 mm to 1.16 ± 0.09 mm, and pitch decreases from 15.81 ± 2.53 mm to 11.59 ± 2.44 mm. Figure 3a also depicts extrusion results when the cooling fan speed is set to 25% and 100%. When the cooling fan speed is lowered to 25%, the PLA filament is too molten to form the necessary helical shape, and when the fan speed is increased to 100%, the PLA completely solidifies within the printhead prior to extrusion.

Lastly, the geometries for muscle groups 9, 2, 11, 12, and 13 are shown in Figure 4. When the printhead temperature and cooling fan speed remain constant at 125 °C, and 75%, respectively, and inlet pressure is increased from 12 psi to 16 psi, the muscle diameter remains relatively stable. At an inlet pressure of 12, 14, and 16 psi, the diameters were found to be 3.58 ± 0.16 mm, 3.24 ± 0.33 mm, and 3.42 ± 0.42 mm, respectively. Fiber thickness only increases from 1.16 ± 0.09 mm to 1.22 ± 0.07 mm when inlet pressure increases from 12 to 16 psi, showing that inlet pressure does not significantly affect muscle diameter or fiber thickness at this pressure range. Artificial muscle pitch also increases from 11.59 ± 2.44 mm at 12 psi to 17.42 ± 5.66 mm at 16 psi. When the inlet pressure is decreased to 10 psi, the material is given too much time to cool within the printhead resulting in a clog. Additionally, filaments that resemble a helical shape can be printed at extreme pressures. Through trial and error, it was discovered that PLA can be extruded into a helical shape below 50 psi; however, at pressures equal to or in excesses of 50 psi, the flow rate within the printhead is too great for the fiber to retain a helical shape. Furthermore, Figure 4c shows that a helical profile can be produced at 16 psi; however, the significant error in artificial muscle pitch at 16 psi indicates that the inlet pressure level is unsuitable for consistent helical artificial muscle production. Therefore, using inlet pressures equal to or above 16 psi is not recommended if repeatable geometries are necessary for a particular artificial muscle application. 

The measured geometry variance caused by single-factor experimentation can be attributed to either the die swell phenomenon or self-gravity. When the printhead temperature increases or the cooling fan speed decreases, the exiting polymer has not solidified and therefore is in a more molten state; allowing the velocity profile to relax and causing the fiber thickness to enlarge or swell [30,35,36]. Additionally, when the printhead temperature or inlet pressure increases, or cooling fan speed decreases, the extruding polymer may form a helical shape but has not solidified significantly to overcome self-gravity. The weight of the muscle then extends the average pitch length as it extrudes from the printhead. Moreover, the self-gravity of the semi-solid artificial muscle during extrusion causes a decrease in diameter since the volume of the muscle must remain constant [30]. Thus, conditions resulting in increased polymer temperatures will increase pitch length and decrease muscle diameter. Therefore, if the geometry is of critical importance to an operator, the printhead should be set to extrusion conditions that promote a more rapid cooling of the polymer when in the presence of a strong gravitational field. It would be expected that self-gravity would have little to no effect in deep space or smaller terrestrial bodies, such as the moon, where gravitational forces are significantly lesser when compared to those on Earth. Furthermore, if an operator wished to increase the diameter of the artificial muscle, the printhead temperature should be decreased, or the fan speed should be increased. To increase fiber thickness or pitch, the printhead temperature or inlet pressure should be increased, or the cooling fan speed should be decreased. 

Lastly, muscle group 2 was found to have an average muscle diameter (3.58 ± 0.16 mm), fiber thickness (1.16 ± 0.09 mm), and pitch (11.59 ± 2.44 mm) closest to that of the ideal geometries of the mandrel flute diameter, depth, and pitch (4 mm, 1 mm, and 8 mm), making the extrusion conditions for this muscle group best suited for printing geometries close to the mandrel geometry. Herein, muscle group 2 will be a representative muscle for all further testing.

### 3.3. Phase Diagram Establishment for Helical Artificial Muscle Formation

**Figure 5:**
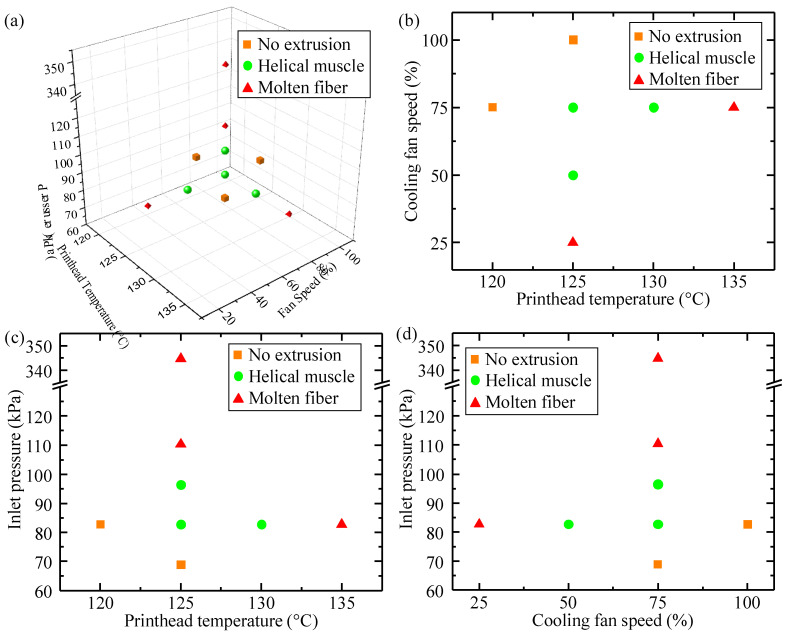
(**a**) Three-dimensional phase diagram of artificial muscle fabrication results at the tested extrusion conditions. (**b**) Sub-phase diagram of artificial muscle fabrication at a constant inlet pressure of 82.7 kPa. (**c**) Sub-phase diagram of artificial muscle fabrication at a constant cooling fan speed of 75%. (**d**) Sub-phase diagram of artificial muscle fabrication at a constant printhead temperature of 125 °C.

To better evaluate the printability and artificial muscle formation, a 3D phase diagram was constructed using the key extrusion conditions and geometry measurement results. As seen in Figure 5a, the combination of key extrusion conditions results in either non-extrudable filaments, helical artificial muscles, or molten fibers unable to retain helical shape.

Furthermore, Figure 5b–d summarize three sub-phase diagrams where the printability and muscle formation are described when only altering two key extrusion conditions and holding the third at a constant. Figure 5b illustrates artificial muscles fabricated at a constant inlet pressure of 12 psi (82.7 kPa). These muscles can be accurately fabricated at printhead temperature/cooling fan speed combinations of 125 °C/50%, 125 °C/75%, and 130 °C/75%. When cooling fan speed is held constant at 75%, as shown in Figure 5c, muscles can be accurately fabricated at printhead temperature and inlet pressure combinations of 125 °C/82.7 kPa, 125 °C/96.5 kPa, and 130 °C/82.7 kPa. Lastly, when printhead temperature is held constant at 125 °C, muscles can be accurately fabricated at cooling fan speed and inlet pressure combinations of 50%/82.7 kPa, 75%/82.7 kPa, and 75%/96.5 kPa, per Figure 5d. Alone, the 3D phase diagram or three sub-phase diagrams could aid an operator’s decision on key extrusion conditions but using multiple phase diagrams to select extrusion conditions may be tedious. 

To simplify printing condition selections, the Brinkman number ($Br_{p}$) and Nusselt number ($Nu_{p}$) of the extruding polymer can be used to create a 2D phase diagram. The Brinkman number is a dimensionless number used to analyze the ratio of viscous heating to conduction heating and is commonly used in describing polymer melt processes [37,38]. The Nusselt number is the dimensionless ratio of convective to conductive heat transfer across a boundary [39,40]. In this study, the Brinkman number describes the influence of the heat transfer caused by pressure-driven polymer melt flow and the printhead temperature. The Nusselt number is used to evaluate the effects of the cooling fan on the extruding artificial muscle. The Brinkman and Nusselt numbers are defined as:

(1) $$Br_{p}=\frac{\eta_{0}u^{2}}{k_{p}(T_{m}-T_{ph})}$$

(2) $$Nu_{p}=\frac{h_{a}(t^{*})}{k_{p}}$$

where $\eta_{0}$ is the zero-shear rate viscosity of the molten polymer, u is the average velocity of the polymer through the mandrel’s flute, $k_{p}$ is the thermal conductivity of the polymer, $T_{m}$ and $T_{ph}$ are the polymer melting temperature and printhead temperature, respectively, $h_{a}$ is the convective heat transfer coefficient caused by the forced convection of the cooling fan across the exposed artificial muscle during extrusion, and $t^{*}$ is the flute depth of the mandrel. To simplify the modeling of the mandrel’s flute, it is assumed that the flute is a narrow pipe with a diameter of $t^{*}$. The convective heat transfer coefficient and average velocity are calculated using the following equations:

(3) $$h_{a}=\frac{Nu_{a}k_{a}}{t^{*}}$$

(4) $$u=\frac{P{\left(t^{*}\right)}^{2}}{32\eta_{0}l}$$

where $Nu_{a}$ is the Nusselt number of air forced over the exposed artificial muscle by the cooling fan, $k_{a}$ is the thermal conductivity of air at room temperature, P is the inlet pressure of the printhead, and l is the helical length of the mandrel flute. In the case of the mandrel used in this study, the helical length is 52.2 mm. The Nusselt number of air can be expressed as a function of the Reynolds number, Re, and Prandtl number, Pr, at room temperature as follows [41]:

(5) Nua=0.3+0.62Re12Pr13[1+(0.4Pr)23]14[1+(Re282,000)58]45

At room temperature, the Prandtl number is assumed to be 0.71 [41], and the Reynolds number is calculated using the following equation:

(6) $$Re=\frac{t^{*}\rho_{a}v_{f}}{\eta_{a}}$$

where $\rho_{a}$ is the density of air at room temperature, $v_{f}$ is the cooling fan speed, and $\eta_{a}$ is the viscosity of air at room temperature. The thermal conductivity (0.132 W/m·K) and melting temperature (155 °C) of PLA, mandrel flute depth (1 mm), and mandrel flute length (52.2 mm), as well as the density (1.161 kg/m^3^), viscosity (184.6 × 10^−7^ N·s/m^2^), thermal conductivity (26.3 × 10^−3^ W/m·K), and Prandtl number (0.71) of air at room temperature, are all known constants and shown in Table 2 [31,41]. The zero-shear viscosity of the PLA build material is unknown; therefore, rheological testing is needed to evaluate the viscous properties of the polymer.

**Figure 6:**
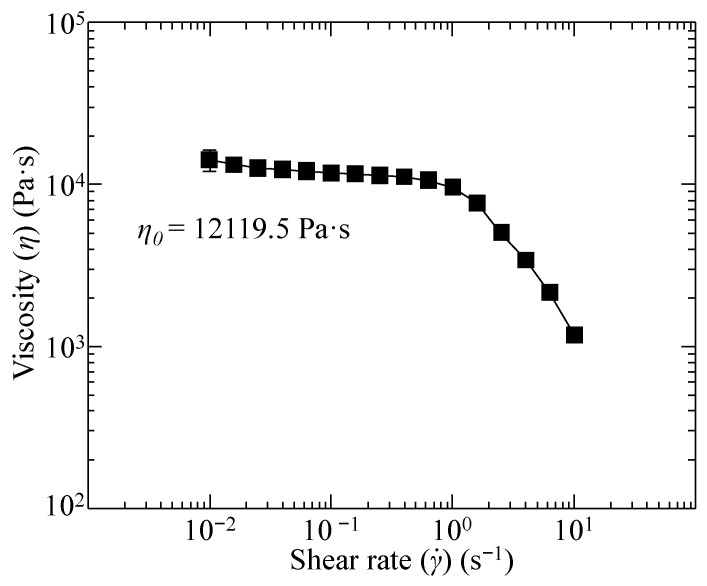
Polylactic acid viscosity as a function of shear rate and the zero-shear rate viscosity at 160 °C.

The measured viscosity of molten PLA at 160 °C is shown in Figure 6. The zero-shear rate viscosity can be extracted from the measured viscosities across a shear rate range of 0.01 to 10 s^−1^ by fitting the data to the Carreau model. The Carreau model is defined as:

(7) $$\eta =\eta_{\infty }+\left(\eta_{0}-\eta_{\infty }\right){\left[1+{\left(\lambda \dot{\gamma }\right)}^{2}\right]}^{\frac{n-1}{2}}$$

where η is the viscosity at a specific shear rate, $\eta_{\infty }$ is the viscosity at infinite shear, λ is the characteristic time,$\dot{\gamma }$ is the shear rate, and n is the power index. Using the viscosity and shear rate data described in Figure 6, the zero-shear rate viscosity, viscosity at infinite shear, characteristic time, and power index were calculated to be 12,119.5 Pa·s, 1.19 × 10^−19^ Pa·s, 0.343 s, and 0.0333.

**Figure 7:**
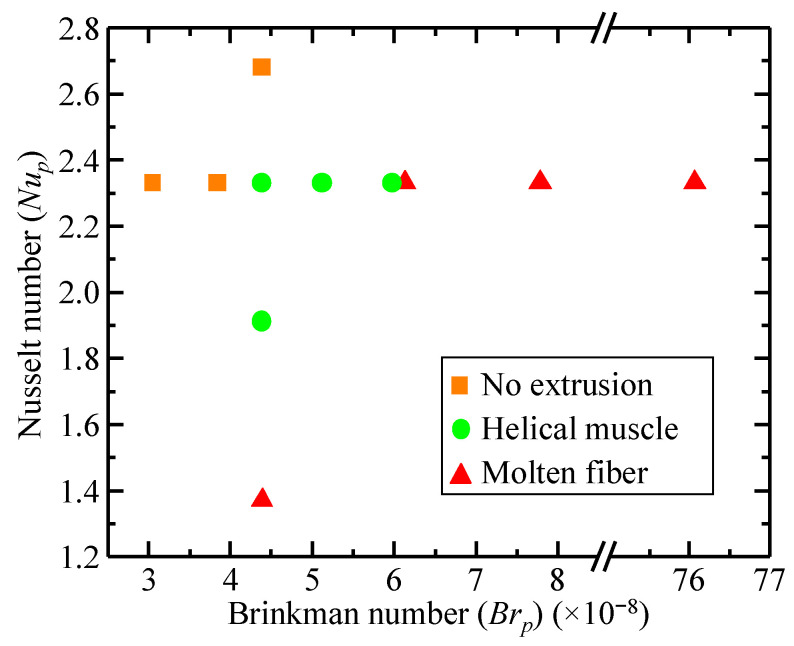
Two-dimensional phase diagram constructed from the Brinkman and Nusselt number of the extrusion process.

Using the zero-shear rate viscosity calculated from the rheological testing, the Brinkman and Nusselt numbers of the polymer during the extrusion process can be calculated. Figure 7 represents the calculated Brinkman and Nusselt numbers of the helical artificial muscle when fabricated using the combination of key extrusion conditions described in Table 1. Using the 2D phase diagram represented in Figure 7, operators of the extrusion system can deduce extrusion conditions resulting in Brinkman numbers between 4.38 × 10^−8^ and 5.96 × 10^−8^ and Nusselt numbers between 1.9143 and 2.3318 will result in adequately formed helical artificial muscles. On the other hand, using extrusion conditions that result in Brinkman and Nusselt numbers outside of these ranges will either produce a molten filament without a helical profile or a clogged printhead.

### 3.4. Mechanical Properties and Surface Morphology

**Figure 8:**
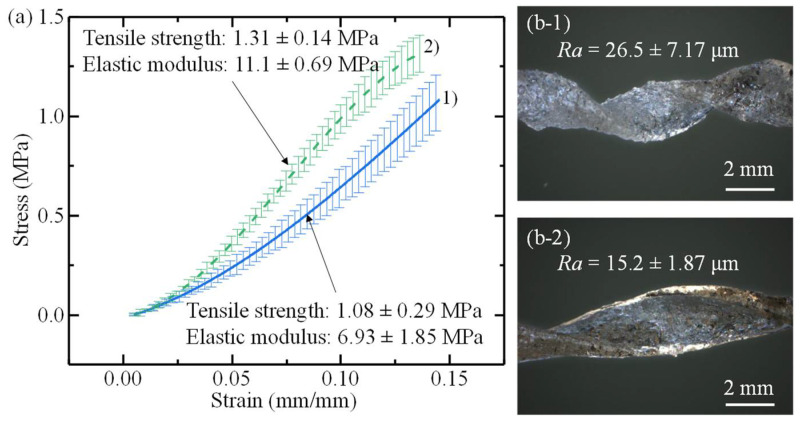
Mechanical and morphology properties of muscle group 2 and 6. (**a**) Stress–strain curves, max stresses, and elastic moduli of muscle group 2 and 6. (**b**) Artificial muscle surface morphology and roughness of (**b1**) muscle group 2 and (**b2**) muscle group 6.

Helical geometry is only one critical aspect of artificial muscle production and cannot drive all extrusion conditions. The mechanical properties of the artificial muscles are also affected by the extrusion process. Figure 8a depicts the stress–strain curve of group 2 and group 6 muscles after calculating their respective average cross-sectional areas. The cross-section area of muscle groups 2 and 6 were found to be 1.23 mm^2^ and 1.07 mm^2^, respectively. Muscle group 6 was fabricated with a lower fan speed than those of muscle group 2, therefore, the muscle group 6 muscles exited the printhead at a higher temperature. The difference in surface morphology and surface roughness is shown in Figure 8b. Figure 8(b1) depicts the surface morphology and average surface roughness of 26.5 ± 7.17 μm for muscle group 2. Muscles from muscle group 6 have significantly fewer surface defects upon inspection and measured average surface roughness of 15.2 ± 1.87 μm, as shown in Figure 8(b2). The decreased surface roughness of muscle group 6 results in a tensile strength and elastic modulus of 1.31 ± 0.14 MPa and 11.1 ± 0.69 MPa, respectively, while muscle group 2 was found to have a tensile strength of 1.08 ± 0.29 MPa and an elastic modulus of 6.93 ± 1.85 MPa. The notch-like structures from the high surface roughness results in crack nucleation sites and reduced mechanical properties for many materials [42,43,44]. Therefore, when fabricating artificial muscles using the helical fluted mandrel printhead, extrusion conditions that affect the mechanical properties and surface morphology of the artificial muscles must be considered.

### 3.5. Contraction Results

**Figure 9:**
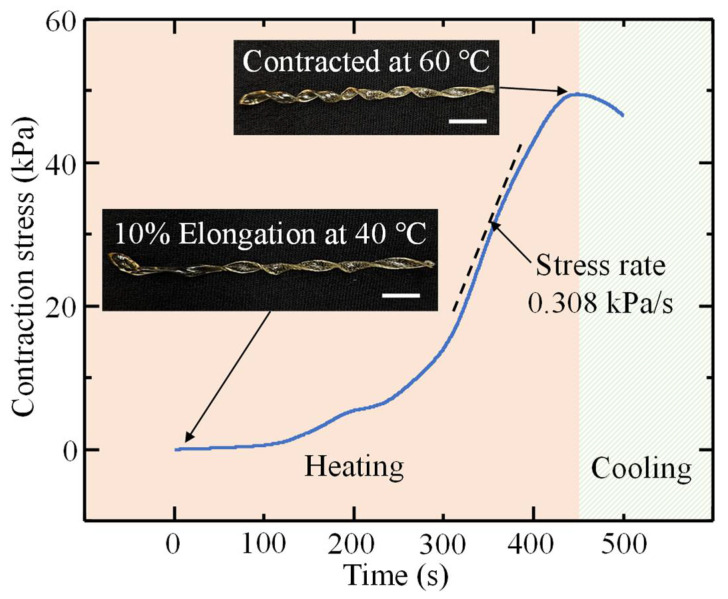
Artificial muscle contraction stress and stress rate over time when heated to 60 °C (scale bar: 10 mm).

Lastly, the helical artificial muscles must contract when stimulated, and the contraction must be quantifiable to determine its viability. PLA is a thermally actuated, one-way SMP; therefore, the muscles must be programmed at or above their glass transition temperature before they can contract [45,46]. The artificial muscles in this study were programmed at 55 °C and elongated to 10% of the original length. Figure 9 depicts the contraction stress of muscle group 2 over time when contracted at 60 °C. With an average cross-sectional area of 1.23 mm^2^, the artificial muscles were able to induce a contraction stress of 49.52 ± 6.01 kPa and an average stress rate of 0.308 kPa/s, validating their ability to contract as artificial muscles.

## 4. Conclusions and Future Work

The formation of PLA helical artificial muscles through a specifically designed extrusion system was evaluated at various extrusion conditions. The geometric measurements and general observation of muscle shape fidelity were used to determine successful printing condition combinations. Depending on the combinations of the key extrusion conditions, the polymer build material cooled too rapidly and clogged the printhead, lacked proper solidification, or fell into a suitable temperature and polymer melt flow range that successfully extrudes artificial muscles. Three-dimensional and two-dimensional phase diagrams were constructed using the results of the 13 printing experiments. After measuring the geometry of all muscles, it was found that increasing the printhead temperature or decreasing the cooling fan speed increases fiber thickness. Furthermore, increasing printhead temperature, reducing the cooling fan speed, or increasing inlet pressure increases muscle diameter and pitch. Additionally, shape morphology and mechanical properties were shown to be affected by the extrusion conditions. Lastly, representative artificial muscles printed with a printhead temperature, cooling fan speed, and inlet pressure of 125 °C, 75%, and 12 psi were programmed and contracted using thermal stimulus. These artificial muscles had a contraction stress and stress rate of 49.52 kPa and 0.308 kPa/s. 

Future work may focus on other extrusion conditions and extrusion through mandrels of differing geometries. Helical artificial muscle extrusion using different SMP build materials will also be tested and compared to previous works. PLA was chosen in this study as a proof-of-concept material to determine whether helical geometry, mechanical properties, and surface morphology could be controlled for a shape memory material. However, the contraction stress of PLA artificial muscles measured in this study is lesser than the two-way SMPs commonly used in artificial muscle fabrication, such as nylon and ethylene-vinyl acetate copolymer. Therefore, artificial muscles will be extruded using nylon and ethylene-vinyl acetate copolymer, and the contraction stresses and strains will be compared to the results published by Haines et al. [17] and Qi et al. [47], respectively. In addition, only three extrusion conditions were tested in this study because they were deemed the most critical for muscle fabrication. Other extrusion conditions, such as the material reservoir temperature, could affect the extrusion characteristics. The extrusion conditions tested in this study should be used to print artificial muscles through mandrel flutes of different diameters, depths, and pitches to better understand which mandrel geometry/printing condition combinations produce adequate helical artificial muscles.

## Figures and Tables

**Table 1:** Extrusion conditions for each muscle group during artificial muscle fabrication.

	Extrusion Conditions		
Muscle Group No.	Printhead Heater Temperature (°C)	Cooling Fan Speed (%)	Inlet Pressure (psi)
1	120	75	12
2	125	75	12
3	130	75	12
4	135	75	12
5	125	25	12
6	125	50	12
7	125	75	12
8	125	100	12
9	125	75	10
10	125	75	12
11	125	75	14
12	125	75	16
13	125	75	50

**Table 2:** Constant values used in Brinkman and Nusselt number evaluations.

Constant	Value	Constant	Value
t*	1 mm	*P_r_*	0.71
*k_a_*	26.3 × 10^−3^ W/m^2^K	*ρ_a_*	1.161 kg/m^3^
*k_p_*	0.132 W/m^2^K	*η_a_*	184.6 × 10^−7^ N·s/m^2^
*T_m_*	155 °C	*l*	52.2 mm

## Data Availability

The data that support the findings of this study are available from the corresponding author (yifeij@unr.edu) upon reasonable request.
